# Promoting Healthier Masculinities as a Suicide Prevention Intervention in a Regional Australian Community: A Qualitative Study of Stakeholder Perspectives

**DOI:** 10.3389/fsoc.2021.728170

**Published:** 2021-12-08

**Authors:** Katherine Trail, John L. Oliffe, Deepa Patel, Jo Robinson, Kylie King, Gregory Armstrong, Zac Seidler, Courtney C. Walton, Michael J. Wilson, Simon M. Rice

**Affiliations:** ^1^ Orygen, Parkville, VIC, Australia; ^2^ Centre for Youth Mental Health, The University of Melbourne, Parkville, VIC, Australia; ^3^ School of Nursing, University of British Columbia, Vancouver, BC, Canada; ^4^ Department of Nursing, The University of Melbourne, Parkville, VIC, Australia; ^5^ Benetas Macedon Ranges Health Centre, Gisborne, VIC, Australia; ^6^ Turner Institute for Brain and Mental Health, Monash University, Clayton, VIC, Australia; ^7^ Nossal Institute for Global Health, Melbourne School of Population and Global Health, The University of Melbourne, Melbourne, VIC, Australia

**Keywords:** masculinity, suicide prevention, men’s mental health, regional and rural mental health, help seeking

## Abstract

Regionally-based Australian men have a higher risk of suicide than those in urban centers, with similar trends observed internationally. Adopting a place-based approach to understanding men’s suicide and harm prevention provides contextual insights to guide localised opportunities for the development of tailored gender-specific interventions. Men in rural Australia are typically portrayed as embodying idealized masculinity–dominant and tough, upholding strength and stoicism in the face of hardship. Such values can increase suicide risk in men by reducing help-seeking. The Macedon Ranges Shire is an inner regional municipality with a population of approximately 50,000 people spanning across 10 regional towns and surrounding farming areas in Victoria, Australia. Understanding the influence of masculinities on men’s wellbeing and help seeking behaviours in a regional context is vital in order to inform effective local suicide prevention efforts. The present research involved in-depth qualitative interviews with 19 community stakeholders (*M* = 49.89 years, SD = 11.82) predominantly working in healthcare and community services including emergency services and education. Using thematic analysis, interview transcripts were coded and themes inductively derived. Stakeholders identified three key areas for understanding suicide risk and wellbeing for local men; 1) localizing masculinities, 2) belonging in community, and 3) engaging men. Findings illustrate that addressing men’s wellbeing in regional areas requires a multifaceted whole-of-community approach. While diverse, local expressions of masculinities were seen as contributors to men’s challenges understanding their emotional worlds and reticence for help-seeking. Of vital need is to provide diverse opportunities for men to connect with others in the region, and offer inclusive spaces where men feel accepted, welcomed and able to meaningfully contribute to the community. Not only will this assist by bolstering men’s sense of self, identity, and mental wellbeing, it may also provide valuable informal inroads to normalizing healthy communication around mental health and seeking mental health care. These findings offer important suggestions for the promotion of healthier masculinities in regionally-based Australian men, which may help to improve wellbeing of these men and their entire communities.

## Introduction

Regional and rural Australian men are at higher risk of suicide relative to those living in urban centers (Australian Bureau of Statistics (ABS), 2020). This is a trend also observed globally (Hirsch & Cukrowicz, 2014). Various factors are thought to contribute to increased rates of regional and rural suicide including reduced access to mental health services, physical and social isolation, increased vulnerability to extreme weather events such as drought, fires and floods, and increased access to dangerous means such as firearms (Hirsch & Cukrowicz, 2014). Given that men are 3–4 times more likely to die by suicide than women globally (Institute for Health Metrics and Evaluation, 2018), research focus has recently shifted to understanding the gendered experience of suicide and the role of masculine norms such as stoicism and self-reliance (Pirkis et al., 2017). Research into masculinities has been guided by Connell (Connell, 1995; Connell & Messerschmidt, 2005), who discusses the ways in which gender is socially defined through everyday patterns of practice dependent on culture and context. Conformity to masculine norms, as defined by Mahalik et al., 2003), refers to “*meeting social expectations of what constitutes masculinity in one*’*s public or private life*” (p. 3). These perspectives provide a model to understand how adherence to masculine norms may differ across contexts and environments, and thus have differential impacts on men’s mental health outcomes (Wong et al., 2017).

Traditionally, Australian rural masculinities have positioned men as independent and tough individuals who exert dominance over their land, and are stoic in the face of hardship (Alston & Kent, 2008). This ingrained stoicism is intrinsically linked with cultural expectations of rurality (Roy et al., 2017; Kaukiainen & Kõlves, 2020) and masculinity (Seidler et al., 2016) both in Australia and overseas, serving as a significant barrier to seeking mental health care for rural and regional men (Cheesmond et al., 2019). While idealized versions of manliness remain to some extent, recent scholarship points to the diversity of masculinities, recognizing that men diversely express their gender, even in rural areas that may appear homogenous (Creighton et al., 2017). For example, Herron et al., 2020) found that within their sample of rural Canadian men, those in distress indicated a desire to talk about their mental ill-health, but felt that their rural environment lacked opportunity to do so, highlighting tension between their agency and existing structures, and the pressure to conform to normative stoicism and self-reliance practices.

Having local social support structures and an increased sense of community can serve as protective factors against psychological distress in rural men (Kutek et al., 2011). McLaren and Challis (2009) found that although depression is a key risk factor for suicidal behaviour, this relationship was not significant among some farming men who reported high levels of social support and belonging. This aligns with the interpersonal theory of suicide, whereby suicidal behaviour emerges in the presence of two key constructs: thwarted belongingness and perceived burdensomeness, in addition to an acquired capability to enact lethal self-harm (Van Orden et al., 2010). Thwarted belongingness, which can occur in the absence of reciprocally caring relationships, can lead to loneliness and isolation. These factors may be exacerbated in regional areas where geography of townships increases physical distance between men and their communities. Understanding factors associated with belonging and connectedness for men in their community may provide vital clues and potential inroads to protecting men from suicide.

Given the aforementioned influence of masculine norms on help-seeking, and comparatively high suicide rates of regional and rural men, particular attention should be paid to specific challenges for men within *their* community (Creighton et al., 2017). Adopting a place-based approach to understanding masculinities and mental health in rural and regional communities will assist in the development of tailored community policy and practice interventions (Cheesmond et al., 2019). Taking a relational view of place, environments can be viewed as dynamic and influenced by the changing mobility of populations as they move in and out of place borders, both daily and across the lifespan (Cummins et al., 2007). Hence, while men may live in small townships, they influence, and are influenced by, the place they live, their workforces, the people they interact with, and external cultural discourses.

The Macedon Ranges Shire in Victoria, Australia is an inner regional area with a population of just under 50,000, spread across 10 regional townships and surrounding areas (ABS, 2018). Of concern to local government and healthcare organisations, the region has comparatively high male suicide rates compared to state rates, and increasing family violence rates and alcohol-related harms for men (Turning Point, 2018; Crime Statistics Agency, 2020; Public Health Information Development Unit, 2021). This has prompted the need for place-based analyses of men’s attitudes and harm-related behaviours, to provide insight into potential factors facilitating these patterns of harm. Place-Based Suicide Prevention Trial Sites (PBSPTs) were established in twelve communities across Victoria, Australia from 2017 to 2020 as part of the State and Commonwealth Government’s suicide prevention strategy. The present study is a part of *The Human Code Project*, facilitated through the Macedon Ranges PBSPT (https://www.benetas.com.au/health-care/macedon-ranges-health/the-macedon-ranges-suicide-prevention-trial-site), designed to determine the extent to which local men and boys conform to dominant masculine norms and the impact of this on men and boys doing and experiencing harm.

While the Macedon Ranges Shire retains an agricultural and industrial focus, the proximity to larger urban centers has brought an influx of population growth in recent years. Many men maintain employment in the nearby city of Melbourne or surrounding townships, commuting out of the region daily for work. The conceptualization of rural masculinities (e.g., Alston & Kent, 2008) focused predominantly on agriculture may not adequately represent men in this community. In this way, the Macedon Ranges may represent many inner regional areas in Australia (and internationally) where changing populations continue to cause demographic cultural shifts as the roles, expectations and expressions of masculinity diversify. As such, place-based research into local masculinities and mental health service provision for men is vital to understand the specific needs of communities, and to be able to develop tailored community driven interventions.

The identification of potential risk and protective factors for men’s wellbeing within a specific community may facilitate upstream suicide prevention strategies focused on cultural change at a community level. This mirrors Australian place-based suicide prevention which seeks to build community resilience through a whole-of-community approach involving government agencies, community and healthcare organisations, and people with lived experience of suicide (Baker et al., 2018; Shand et al., 2020). Stakeholder views have been sought previously in male suicide prevention (e.g., Grace et al., 2018) and can aid in the development of practical strategies and increase the likelihood of implementation of such interventions (Wolk et al., 2018). Using in-depth qualitative interviews, the present study aimed to understand stakeholder perspectives of how local men enact masculinities, and how masculine norms influence men’s wellbeing in a regional area. The findings will inform *The Human Code Project* and facilitate the development of healthy-masculinity focused initiatives in a regional community. More generally, the current research offers an example of localized qualitative enquiry into masculinities and men’s wellbeing that may provide a guide for future community-based suicide prevention research.

## Methods

### Study Background

*The Human Code Project* was commissioned by the North Western Melbourne Primary Health Network through the Macedon Ranges Place-based Suicide Prevention Trial Site with the purpose of identifying key data to inform subsequent program development for the promotion of healthier masculinities within the Macedon Ranges Shire. This project focused on developing local research knowledge of the attitudes and behaviours of men and boys in the Macedon Ranges, in order to contribute to the identification of healthy masculinity-focused approaches to reducing male suicide. Integral to the project was the input of local voices, and the use of local resources and skills to ensure the successful implementation of evidence-based initiatives within the community. A Project Working Group was established in September 2020 comprised of key local partners and stakeholders including council, local healthcare providers, suicide prevention and family violence networks, Men’s Sheds, local sporting clubs and health promotion organisations, responsible for oversight and community input into the project. Note that the Project Working Group did not include any members from the study funder. *The Human Code Project* ran from September 2020 to August 2021 and involved a community-wide survey, interviews with community stakeholders, and interviews and focus groups with local men and women. The present study utilizes data from interviews with community stakeholders. This research was approved by The University of Melbourne Human Research Ethics Committee (2057593).

### Study Sample

Nineteen stakeholders (52.6% female) ranging in age from 33–81 years-old (*M* = 49.89, *SD* = 11.82 years) working in the Macedon Ranges Shire completed individual interviews. Stakeholders consisted of healthcare and health promotion professionals (*n* = 8), community service, law enforcement or sports staff (*n* = 8), and education staff (*n* = 3). Average length of interviews was 45 min. A minimum sample size of *N* = 15 was determined a priori on the basis of the finding that 12 in-depth interviews is likely to result in data saturation (Guest et al., 2006). The sample was extended to *N* = 19 in order to obtain adequate scope across stakeholder professions on direction of the Project Working Group.

### Data Collection

Participants were recruited through the professional networks of the Project Working Group. Community groups, local service providers, organisations and individuals that had expertise or experience working with boys and men were determined by the Project Working Group. Organisations and/or individuals were then contacted by the research team via phone or email to explain the study aims and interview process. Those who expressed interest in participating were provided with the study information statement and consent form. Stakeholders were sought from a range of community sectors including health care and emergency services, LGBTIQA+, youth and older people’s community services, community sport, law enforcement, and education to ensure breadth of experience in working with local men and boys across age brackets. Participants were offered a $30 reimbursement via EFT transfer as recognition of their time. Following the provision of informed consent, interviews were conducted virtually using Zoom videoconferencing software (Zoom Video Communications Inc., 2019). Conducting interviews remotely via Zoom was beneficial in that it facilitated ease of data collection and overcame geographical barriers to participating in the research, particularly given the researchers were not located in the area of interest. Further, the online setting lowered the time commitment for participants who were often completing their interview during working hours. Of the 19 interviews, only one had notable disruptions due to an unstable internet connection. This was quickly overcome by using Zoom’s dial in feature, allowing the interview to continue smoothly. Unstable internet connection and/or lack of access to internet however may be limitations of using videoconferencing for qualitative interviewing, particularly when working in regional and rural communities where internet shortages may be more common. One disadvantage of using videoconferencing was a higher rate of disruption during interviews due to other people or external noise, however this was managed by the researcher asking participants to ensure they were in a quiet and private space for the duration of the interview, and by being flexible and adaptive if interruptions were to occur. Benefits and limitations of using Zoom mirrored those discussed recently by Oliffe et al., 2021). Overcoming the above limitations, Zoom was a successful and acceptable method for participants and researchers in this study.

Interviews followed a semi-structured interview schedule with key questions including “*What do see as the biggest risks to the health of boys and men in the Macedon Ranges community?*”, “*How does the Macedon Ranges community influence the development of masculinity for men and boys in the region?*”, and “*How could organisations in the Macedon Ranges help reduce the likelihood of men experiencing a mental health crisis, including suicide attempt?*” (See supplementary materials for complete interview schedule). The first interview was conducted jointly by researchers KT and SR. The remaining 17 interviews were conducted by one 24-year-old female researcher KT (BSc Hons) who was supervised by SR (PhD MPsych) who has significant experience conducting qualitative interviews with men. 16 interviews were individual and one involved two participants upon their request to be interviewed together. The researchers were not local to the area of interest and therefore had no pre-existing relationship with the participants or specific culture being discussed. The researchers were guided by a theoretical understanding of rural Australian masculinities (e.g., Alston & Kent, 2008), and preexisting knowledge of concerns in the area (e.g., high male suicide rates) which may have influenced perceptions of the area and subsequent data. KT kept memos during the data collection process noting any assumptions or biases that presented for discussion with the wider research team. Demographics (age, gender identity and professional sector) were obtained at the beginning of each interview.

### Data Analysis

Data were analysed according to qualitative research techniques and reflexive thematic analysis (Braun & Clarke, 2006, 2019). Audio recordings of interviews were transcribed verbatim, and checked for accuracy by KT. Transcripts were deidentified and participants allocated a unique code. Data familiarization was firstly gained by reading the full data set in depth. Basic concepts were then identified inductively, and data segments were assigned to descriptive codes in NVIVO-12 qualitative software (QSR, 2018). Cross-coding of two full interview transcripts was undertaken by two authors (KT, MW) with incongruences discussed and consensus reached to ensure consistency in the coding analytics. Initial codes were then organized into broad themes to assist with organising the data to allow for further analyses. Iteratively, codes were subsumed and weighted to inductively derive beginning insights. For example, initial codes used descriptive labels that reflected direct meaning from participants (e.g., “*Banter and taking jokes as a key part of masculinity*”, “*Driving related to masculine identity*”) and these codes were subsumed into categories representative of broader patterns in the data such as ‘*Regional* “*blokey*” *culture*’. In further analyzing the coded data, themes and subthemes were developed, defined, and re-named to pre-empt the discrete findings. For example, ‘*Regional* “*blokey*” *culture*’ was subsumed into the wider subtheme ‘*Embodying local masculinity*’*.* These analytic processes included researcher meetings (SR, KT, JLO) to discuss the data and select representative illustrative quotes, which occurred regularly in the drafting of the findings and current article. Masculinity (e.g., Connell, 1995) and gender and place (Massey, 2008) frameworks were drawn upon to theorize and contextualize the findings within the men’s mental health literature.

## Findings

Three overarching themes were derived from the interview data; 1) *Localizing masculinity* 2) *Belonging in community*, and 3) *Engaging men*. Within each theme, inroads for intervention as suggested by stakeholders are discussed to provide potential direction for making meaningful change. Themes and subthemes are presented in Table 1.

**TABLE 1:** Summary of themes and subthemes.

Theme	Sub-theme
Localizing masculinities	Embodying local masculinity
	Restricting mental health help-seeking
	Breaking the masculinity mould
Belonging in community	Community connectedness as a requisite to wellbeing
	Linking social disconnection to distress
	Creating opportunities for connection
Engaging men	Authenticity forefronting intervention
	Tailoring language, program content and delivery

### Localizing Masculinities

The first theme, localizing masculinities discusses stakeholder perspectives about regional men’s expressions of masculinity comprised of three subthemes. First, ‘*Embodying local masculinity*’ summarizes the culture prevalent in the area in regards to enforced gender roles and the continued influence of masculine norms such as stoicism and toughness. The impact of these masculine expressions on men’s mental health is then detailed in the subtheme ‘*Restricting mental health help-seeking*’*.* Despite the traditional culture referenced, stakeholders noted experiences of men who step outside of the perceived norm, and the challenges they may face in doing so were discussed in the subtheme ‘*Breaking the masculinity mould*’. Here, strategies outlined by stakeholders as ways of promoting and sustaining healthier masculinities in the area are presented.

#### Embodying Local Masculinity

Stakeholders referenced a somewhat traditional and conservative Australian culture in the Macedon Ranges, where gender roles were prescriptive and policed. Regional men were seen as needing to conform to expectations of being the main family provider and protector figure. While individual variability in whether men fulfill these roles was accepted, the lack of diversity and the limits to role modelling regarding alternate expressions of masculinity (and minimal opportunities for men to step outside of these roles) was seen as a contributor to pressures faced by men of all ages:

“Growing up in what is essentially a small country town I think can be hard for men, in that sense of, you need to stick to kind of traditional male roles.” (Female, 40–45 years).

Referencing the ‘*blokey*’ culture of the Shire, some stakeholders spoke of masculinity being intertwined with sport, alcohol and drug use, and risk-taking behaviours such as hazardous driving. At the same time, conversations between male peers related to personal difficulties were seen as rare–and perhaps unlikely. Instead, friendship and ‘*fitting in*’ relied on an ability to ‘*take a joke*’, including displays of toughness, bravado and care-less expressions. These outward displays of masculinity were linked with maintaining a sense of power:

“And that’s about getting your license, having a car, having kind of power, I suppose you would say, and feeling like you have achieved something. Whereas I find the more female identifying people don’t. That’s not as much as part of their identity.” (Female, 55–60 years).

#### Restricting Mental Health Help-Seeking

The influence of traditional masculine norms such as stoicism and emotional restriction on men’s help seeking behaviours was apparent across stakeholder interviews. The majority of stakeholders referenced men’s challenges in displaying or talking about their emotional states. This was guided by a cultural attitude of ‘*she’ll be right*’ stoicism which was seen to be enhanced in the regional context:

“Their response is just informed by this picture of masculinity, which is like a squinty eyed, grit your teeth, in English we’ll call it stiff upper lip, that this picture of an unfeeling stone, which is I think brittle, not tough.” (Male, 50–55 years).

Stakeholders described men in the Shire as often having low emotional awareness, with many lacking the skills to talk effectively about their emotional lives. For the most part, this was framed as a learned state whereby men (and boys) were socialised from a young age to refrain from vulnerability and expressing emotions, and therefore lacked opportunities to develop skills to identify and communicate diverse emotional states:

“They’re less likely to seek emotional support or psychological support than women. And that is just a construct of how they’ve been taught. I guess through that, not just being raised, but through culture that, “he’d just pull it together and keep going”. Women traditionally talk to each other, they“re supposed to need support, whereas men are supposed to be the supporters.” (Female, 45–50 years).

Stakeholders reflected that these characteristics may be amenable to change. Increasing awareness and education efforts for men and boys regarding identifying and managing emotions was seen as a potential way to support better self-management of mental health and recognition of distress in peers:

“You’ve actually got to start educating these guys on the language. You’ve got to bring it right back, right, a massive re-build. And actually go, “This is what you say. This is how you feel.” “Hey, this might add up to thoughts of suicide, yeah? Maybe you need to pay attention and listen to what they’re talking about, and acknowledge it and hear it.” And then … talk about being a good listener, which is really important.” (Male, 45–50 years).

Despite their perceived inability to talk about their mental health, men were sometimes seen to rely heavily on their female peers for emotional support, nurturing and managing distress. This may suggest that men confide their concerns to a trusted person, especially if this person is a partner. Fostering acceptable and comfortable environments between male peers could be an important step to building open communication channels. Educating, role modelling and supporting men to have open conversations about mental health with their male peers was seen as a vital part of suicide prevention:

“They’re life-saving conversations. ‘You’ll be right, she’ll be right,’ no. But, ‘I love you. I’m worried. You should go and see a doctor. I’ve been to see a counselor.’ And they’re life-saving conversations.” (Male, 45–50 years).

#### Breaking the Masculinity Mould

While stakeholders reflected on a traditional picture of masculinity being prevalent in the Macedon Ranges, alternate ideas of what it means to be a man were increasingly apparent within the community. For example, one stakeholder described his understanding of masculinity as one that involves emotional awareness and empathy, and rejected typical norms of stoicism and dominance:

“I tend to find a trait of masculinity to be more empathetic and more caring and more outward reaching to try and help people or check in on people rather than inward facing and trying to bottle up things and fall into those very typical type gender roles of being a provider and the dominant figure in relationships and society.” (Male, 30–35 years).

Some stakeholders spoke of experiences with men and boys who are increasingly questioning the benefits of subscribing to the restrictive masculinity with which they are presented. Young men in particular were seen as drivers of change as they look for alternative ways to express their masculinity. This decision to deviate from the norm presents challenges for men as they may struggle to maintain a sense of belonging while engaging in and promoting healthy behaviours and traits that challenge outdated masculine stereotypes:

“There are a lot of young men who are questioning those … outmoded, harmful stereotypes and models of masculinity, who want more, who may look at older men, their fathers and grandfathers, and who actually feel some compassion … young men who are looking to redefine a new kind of masculinity, a new way of doing manhood and boyhood. And I feel there’s quite a lot of openness and some real resilience, it’s not easy to challenge those kinds of societal norms and it’s not easy to stake out a place.” (Male, 50–55 years).

Affirming men to continue to expand their understanding of what it means to be a man in the Macedon Ranges, and actively supporting those who do display alternate pictures of masculinity was seen as a vital way to ensure those behaviours and traits are modelled and reinforced within the community. Empowering men to stand up, be involved and act as positive community role models were articulated as ways of assisting in creating meaningful grass roots community change. This was viewed as particularly pertinent when trying to create change around behaviours that predominantly are seen in men, for example in relation to family violence:

“And I think that needs to continue, to keep having that conversation and getting more Male Champions of Change, for want of a better term to actually stand up. If we talk about domestic violence, family violence, there’s been a lot of female leaders in the community standing up, we need more males.” (Male, 50–55 years).

Importantly as articulated above, the expression of masculinities that contest dominant (and damaging) norms may jeopardize the need to fit in with the peer group, a phenomenon which seems to be heightened in the regional context due to limited social circles available. Social standing, which likely pertains to complicity in sustaining and/or conformity to the norm, was viewed as an important consideration by some stakeholders when identifying community role models who are likely to have the most impact:

“How do you find the young men who are courageous enough, but also have enough social clout in a way, to be the ones to stand up? Because I think that’s often what it is about, is having legitimate, believable, real people in the community that are respected and well regarded.” (Female, 40–45 years).

Characteristics such as kindness, empathy, self-awareness, respect and open communication were viewed as important to uphold and promote via community role models who actively exhibit these characteristics and are celebrated for doing so. These behaviours were more linked to normative femininities, which may explain some men’s reluctance to be seen as embodying these ‘softer’ sides. As highlighted below, men who exhibit these qualities within the accepted framework of masculinity might be most useful in shifting norms to promote and sustain healthier masculinities:

“Men that are self-aware and open, welcoming and embracing. I guess to tie into some of the typical masculine rhetoric, a good bloke type narrative but still someone that can upend the very negative aspects of masculinity.” (Male, 30–35 years).

### Belonging in Community

Across interviews, stakeholders referenced men’s social relationships, community connections and desire to fit in with the norm as essential to their wellbeing, represented here in the theme *belonging in community.* First, the benefit of strong social ties for men and boys is discussed in the subtheme ‘*Community connectedness as a requisite to wellbeing*’. The impact of impeded belonging is then articulated in the subtheme ‘*Linking social disconnection to distress*’. Stakeholders suggestions for ‘*Creating opportunities for connection*’ as a way of improving men’s wellbeing are outlined.

#### Community Connectedness as Requisite to Wellbeing

Many stakeholders highlighted social and community connectedness as requisite to wellbeing for men in the region. Men were seen as benefiting from strong social ties within the community when they were able to muster relationships involving a reciprocity in giving, as well as receiving support:

“I think it is about being involved, being part of someone, a family, a community, a something, so that you are, the men are important, they’ve got a need, they’ve got a reason to live, whatever that might be.” (Female, 60–65 years).

Participants reflected that a sense of belonging, purpose and acceptance within the community, stemming from strong social networks, helped protect from psychological distress and suicide risk in a two-fold way. First, being part of a social network (whether family, peer and/or community group) provided men with a way to engage in healthy communication and relationships, often while partaking in activities that were enjoyable and enriching. Second, trusted social connections offered opportunities for a vital link to supports for men when needed. As men may be hesitant to connect with formal community-based mental health services, strong social networks could provide intermediate informal support and, with the right mental health awareness and help-giving, allow for a trusted link to more formal services to be made when needed. As noted by a male stakeholder, fostering a “*real sense of community … where people look out for each other … will really help to create the glue of the society that people can actually feel supported” (Male, 30-35 years)*.

Men were perceived as having few opportunities for social connection, and/or were less likely to prioritise social connectedness relative to female peers. Although many factors may contribute to men’s difficulties creating and maintaining social connections, stakeholders brought to light several key contributors. First, men’s work and family commitments could pose barriers to prioritising their social life. This may be particularly pertinent in regional areas where a large proportion of the workforce are based outside of their townships, contributing to long commuting hours that keep local residents away from their families and communities for longer. Second, men were perceived as taking less initiative (compared to women) in leading or attending social or community groups. While sporting clubs were something of an exception and viewed as an integral part of the community providing men with vital opportunities for social interaction and support, few alternate avenues for men to connect with the community were mentioned by stakeholders. In particular, the challenges for younger men in finding time and avenues for social connection with peers were highlighted:

“I think there’s just not the opportunities for some of the younger people in town to actually contribute to the community. They go to school outside of town, they come home, they sleep (at) home. They don’t have that opportunity within their community to even catch up with lots of friends or peers or other people in the community.” (Male, 50–55 years).

#### Linking Social Disconnection to Distress

The effect of limited opportunities for local social connection was apparent to some stakeholders who referenced isolation and loneliness as contributing to distress in the men they saw and worked with. Specifically, the impacts of social disconnection may be heightened for those residing outside of townships in the Macedon Ranges. These men were deemed to have less opportunities to interact with other members of the community, and experienced inherent challenges with accessing social support services:

“If they feel like they can’t talk to people or reach out or just become part of the community, I think men often feel on the edges of community unless they’re involved in sports … for older men, things like Rotary or Lions, I think they often feel a bit alone and I guess that’s really tricky.” (Female, 40–45 years).

Social isolation was viewed as a place-based concern for the community as difficulties with transport and physical distance between town centers contributed to men’s resistance and/or inability to engage with social and community groups. In addition to the structural barriers of transport, the regional context could impede men’s sense of belonging. This was discussed in the context of difficulties finding like-minded male peers in a small community. The expectations for how men are, and how they behave, were significant here, especially in relation to age. For those young men who visibly operated outside of the accepted or mainstream ideal of what it means to be a man, there were “*limited options for how young people can see themselves and who they can be, and where they can connect, and belong.*” *(Female, 40–45 years*).

#### Creating Opportunities for Connection

In order to increase men’s social and community connectedness, stakeholders suggested multiple avenues to provide opportunities for men to both make, and maintain social connections. The need for a diverse range of appealing activities and social groups for men was communicated by stakeholders, suggesting a need to expand beyond the existing opportunities and provide more spaces for men with varied interests to feel welcomed, included and able to contribute. Existing groups such as local sports clubs, Men’s Sheds, Rotary and Lions clubs were seen as a vital and beneficial part of the community, offering a place for some men to gather and connect over shared interests, concerns or activities. Stakeholders reflected that informal gatherings and spaces that provide a place for men to talk with peers help to reduce the stigma of discussing mental health concerns. The extension of these models into wider community structures that appeal to other demographics, and age groups, could provide an inroad to connecting more men with their community and fostering the development of healthy social relationships:

“I think we need to broaden our range of opportunities to offer people. So Men’s Sheds are great, but they will apply to a number of people. Organizations are great too, but a lot of it is, I just think about getting to know the person and really working out what they want. What’s going to be beneficial for them rather than us as organizer or for us offering situations for them to come to” (Female, 60–65 years).

Having strong social connections was not only seen as a potential protective factor against psychological distress, giving men a sense of belonging, it was also envisioned as a way for men and boys to connect with services if and when distress occurred. Trusted peers and friends can not only provide informal emotional support, but often are able to encourage or recommend professional support if they have the knowledge and skills to do so. In particular, stakeholders stressed the importance of boys and men being able to choose their potential support figures; who they would go to if they needed emotional support, before reaching the stage of needing crisis support. Here, introducing health promotion messaging around maintaining mental wellbeing, promoting healthy peer relations particularly male-male relations, and identifying key support figures may be a vital step to increasing men’s comfort and willingness to access services:

“I think this whole education thing (is important) about what is good mental health? Teaching people about how to be well and stay well … I think it’s really important with young people to get them to identify who they would talk to if they were struggling, before they struggled, not when they’re struggling … And what it does is it then makes for a much more resilient young person, who makes a much more resilient older person, which then hopefully makes them produce resilient young children.” (Female, 60–65 years).

### Engaging Men

Across interviews, stakeholders highlighted various ways in which local programs and initiatives can better assist men, both by reaching out and engaging with those who are resistant to seek help, and by better responding to those who do access services. Successful approaches were seen as needing to center around men’s individual experiences, strengths and work within a positive masculine framework, outlined in the subtheme ‘*Authenticity forefronting interventions*’. The second subtheme ‘*Tailoring language, program content and delivery*’ then articulates the specifics of how programs may seek to achieve this through tailoring language and content for men.

#### Authenticity Forefronting Interventions

Stakeholders highlighted the need for a person-centered approach when working with men and addressing male distress. Recognition of diversity within men, alongside the specific needs, experiences and expectations of each man when accessing services or programs was seen as an important base by stakeholders. Programs and services that engage men’s agency by lobbying them to contribute their skills and communicate their wishes, built on a philosophy of “doing with and not doing to” were framed as key to successful programs and community change.

“Everybody has skills and strengths, and I think we need to give them opportunities to share those. And once again, it goes back to what opportunities are we putting out there for people to put their hand up and say, ‘Yeah, I can do this. I can be part of this’.” (Female, 55–60 years).

In addition, stakeholders discussed taking into consideration the activities, spaces and groups that men already engage and interact with, and utilising these to create meaningful change in a way that feels familiar and comfortable for men:

“It’s just creating things that men feel comfortable with, working within masculinity to change it, if that makes sense. So trying to reach men on their level and then shift the parameters a little bit at a time, I think is important.” (Female, 45–50 years).

Given the importance of sporting clubs in the region, stakeholders saw a key opportunity for club leaders to advocate for mental health help-seeking and provide support to men in the community in a space that is familiar:

“Before a game, break the ice and say, “Listen, anyone suffering from mental health or they think need to talk to somebody, come over to the club rooms anytime. We’re here for you.” Just get out there and say it on loudspeaker. Then as a club, you’re advocating that it's okay to have some problems.” (Male, 50–55 years).

Some stakeholders discussed the importance of actively promoting a safe space for vulnerability through open communication and modelling of healthy behaviours by program leaders. When men are provided with a “*safe, controlled, supportive environment*” *(Male, 50–55 years)* they are more likely to be willing to engage in discussion around mental health which may help alleviate some of the stresses caused by pressure to manage distress on their own, and allow services to respond to the distress accordingly:

“Of course they’re not going to talk about it, unless you create the right place and space for them. These services, in all fairness, can’t do a whole lot if the bloke’s not admitting what’s going on for them.” (Male, 50–55 years).

The value of championing lived experience as a way to connect and engage with men was discussed by stakeholders. By norming experiences and struggles, authentically sharing lived experiences was seen as a way to create meaningful connections, provide support, education and hope to those who may be going through a similar situation:

“With something as important as mental health, and so much fantastic lived experience where people have come out the other end a better person for that experience and got through some really tough times, having them share that experience I think’s really important. If we can try and promote those people to, I suppose to tell their story and what they went through, what worked for them, what didn’t work for them, because someone will always be able to relate to that, and someone else will be able to relate to someone else.” (Male, 35–40 years).

#### Tailoring Language, Program Content and Delivery

Language was seen as a key tool in the provision of a men-centered approach, with stakeholders arguing for an increased awareness of language that engages with men and allows them to relate to, if not lead, the conversation. Men were seen to benefit from a direct, straightforward conversational approach that provides information in a way that is clear and easy to understand:

“Changing language about how we talk about things is really important, including men in the conversation, because I think a lot of the time they’re not included and it’s seen as the women’s responsibility to fix emotional problems.” (Female, 45–50 years).

Additionally, removing explicit mention of ‘mental health’ and other associated words from campaigns and programs may help men engage better by minimising the stigma felt when discussing their emotional concerns. Instead, framing the conversation around stresses and management experiences or behaviours that men recognise and relate to might make them feel able to engage with the conversation, and open doors to continue discussion and education around mental health and wellbeing:

“If you had … “Are you suffering mental health? Go and see a doctor.” I’d be thinking, if I’m reading that, everyone’s looking at me. Why not target male toilets in the public facilities with advertisment? Without saying to them, “This is to address your mental health.” Like, “Have you been feeling low lately? Have you thought about joining a local sporting club?” (Male 50–55 years).

Normalising behaviours in a non-judgmental format was also seen as a key component to engaging men. Being upfront and openly talking about struggles and problematic behaviours may help to destigmatise and validate men so that they engage the conversation and consider accessing appropriate services. In addition to educating men about the negative impacts of their behaviour for both themselves and others in a non-judgmental forum, facilitating their skill development to navigate similar situations in healthier ways in the future may help to prevent harm to both themselves and others:

“If I come back to domestic violence, a lot of the language in domestic violence is about women. And I think it’s the men that actually need to be targeted in those sorts of campaigns. So even just calling it violence against women or domestic violence, it’s violence men do. I think we need to actually really start speaking the truth about those things, because often perpetrators of that sort of violence are people in crisis. So if the support is given to them, then it helps.” (Female, 45–50 years).

Using positive framing and leveraging men’s strengths was seen as a key way to increase engagement in mental health promotion efforts. In particular, reframing masculine norms may show men how they can better engage in healthy and open communication, without feeling threatened by the perceived loss of masculinity. For example, framing vulnerability as strength might help men to feel more comfortable in standing up and seeking help, if as a result they are viewed as courageous by their peers and community:

“Vulnerability is the ultimate sign of courage. So, men want to be seen as courageous. So, utilizing the things that men want to be seen as, but showing the softer side of that, that actually is a strength.” (Female, 40–45 years).

Additional strengths that could be threaded into communication with men in the context of service delivery or programs include men’s strong sense of care and protection for their loved ones, which may motivate them to look after their health for those around them. Furthermore, there was a sense that the social pressures which have caused men to remain stoic have also built resilience which can be leveraged as hope. Finally, stakeholders noted a willingness of many men to learn and put in the work necessary to solve problems and create positive change, once given the space and time to do so:

“I think often there is that capacity to … do the work. So they go, “Okay. What do I do?” There is a real eagerness to work on some of those things, and … once you’ve set up a safe space, once someone really gets a bit of trust and understands that you are genuine, I think there’s a huge capacity [for change].” (Male, 50–55 years).

## Discussion

The present study adopted a place-based approach to male suicide prevention by describing the challenges and opportunities that exist for men’s wellbeing, and the role of masculinities in these, within a regional Australian community according to local stakeholders. The three themes outlined represent key areas of consideration in increasing wellbeing of regional men and boys in order to reduce suicide risk. The contribution of local expressions of masculinity to men’s social relationships, wellbeing and help-seeking behaviours were key challenges for many stakeholders, reflecting a need to increase visibility of positive expressions of masculinity through promoting healthy role models for boys and men. Evident in stakeholder accounts was the importance of bolstering men’s sense of belonging in community through providing wide-ranging opportunities for social connection and contribution. Practical strategies for engaging with men through gender-sensitive approaches and a strength-based framework were endorsed by community stakeholders, giving key insights for the development of future community programs and initiatives.

### Localizing Masculinity

The characteristics of masculinity spoken of by stakeholders suggest that many men in a regional community adhere to masculine norms of stoicism and emotional restriction, and face pressures to align with idealized masculine roles of being the main household provider and protector figure. Stoicism was much cited in the interviews as a characteristic of men that hinders their ability to cope with and seek help for mental health concerns, as per previous literature (Seidler et al., 2016; Cheesmond et al., 2019; Kaukiainen & Kõlves, 2020). Most stakeholders positioned regional men as especially unlikely to talk about mental health concerns, displaying high emotional restriction or lacking the literacy, modelling or skills to adequately discuss emotional distress. While many men may face challenges in disclosing mental health concerns and expressing emotions that threaten masculine status (River & Flood, 2021), recent research points to the importance of recognizing the agency and structural contexts in which men’s talk does and does not occur (Chandler, 2021). Stakeholder (and possibly broader community) assumptions around men’s disclosure and communication skills may underestimate the power of place for silencing men in this regard. As illustrated by the findings, stakeholders are aware that men may better respond without explicit mentions of “mental health”, and yet an assumption remains that men should be persuaded to talk openly about just that. Listening instead to the men who *are* communicating about emotional stress via their preferred disclosure methods and amplifying this may assist to shift the notion that “men don’t talk”.

Men’s disclosure patterns in times of stress vary both between and within men responding to diverse time points and contexts. As argued by Schwab and colleagues (2016), in emotional disclosure men can present identities that are both resistant and complicit with traditional masculine norms as they navigate the conflicting needs of social connection and presenting as appropriately masculine. Healthcare workers and broader community members should aim to move away from the conceptualization of individual men as either “masculine and emotionally repressed or sensitive and open” (p. 305), and instead be attuned to the ongoing movement between these states. Importantly, recognizing the fluctuation of men’s willingness to disclose may provide markers of growth and resistance to change that can allow listeners reflexivity in responding to men’s changing needs (Schwab et al., 2016). Consequently, as mentioned by some stakeholders, communities should seek to understand the contexts in which men *do* feel comfortable to share their experiences, and what it takes to create safe spaces to affirm and work with men’s disclosures. As argued by Herron and colleagues (2020), men’s perception of the lack of appropriate spaces in regional and rural areas may significantly limit the public community-based sharing of vulnerabilities.

The importance of context in men’s perceived inability to talk about their mental health was highlighted by the findings that men were seen to rely heavily on their female peers for emotional support, nurturing and managing distress. This may suggest that men confide to talk about their concerns with a trusted person, especially if this person is female. The reasons for this were unclear, but may relate to men’s concerns regarding becoming distressed in the company of male peers and perceptions that this may be ridiculed, unacceptable or intolerable, or that their disclosures may not be held with appropriate care or respect (McKenzie et al., 2018). Further, given stakeholder observations of male peer relationships centering around banter and humor, it is likely that there is limited space for expressing vulnerability within such friendships. Recognizing the diversity of men’s patterns of making and maintaining social relationships and understanding the ways in which men do pursue emotionally supportive relationships may help to promote healthier connections among men (McKenzie et al., 2018).

Despite the above concerns, diverse expressions of masculinity were apparent in the region, according to stakeholders, with a perception that younger men tend to value openness, resilience and compassion. The specific contributors to the development of these more positive expressions of masculinity however were not clear to stakeholders, and further research aimed at understanding how this group of men have formed these values may be beneficial. As highlighted by stakeholders, moves to reject rigid masculine norms longstanding in the community may result in men being othered, with increased difficulties finding like-minded peers to form strong social networks with. In a somewhat policed community, existing within the dominant masculine norm allows men to maintain a sense of belonging, paramount for wellbeing. However, given that adherence to masculine norms is linked with increased distress and harm behaviours (Wong et al., 2017), men should be encouraged and supported to embody healthier masculine states and ways of interacting. Young men in particular may be experiencing confusion around their roles and what is expected of them, which can often result in ‘masking’ as more manly (Vandello & Bosson, 2013; Wilson et al., 2021). Increasing men and boys’ confidence to recognize and reject harmful norms, and to be their authentic in their own masculine identities may decrease anxiety related to perceived threats to manhood, and promote positive health behaviours. Utilizing this positive masculinity framework, (Wilson et al., 2021), young men and boys can be supported into more positive psychosocial trajectories through facilitating connectedness and authenticity. Strategies aiming to change attitudes and promote healthier masculinities should consider how to do so in a way that allows men to maintain community belonging and support, to reduce further ostracization and isolation.

### Belonging in Community

Strong social connections fostering a sense of belonging were seen as a key pillar of men’s wellbeing and protection against suicide, mirroring much existing literature and theory (McLaren & Challis, 2009; Kutek et al., 2011). Stakeholders referenced men’s disconnection as a contributor to loneliness and isolation that they viewed as precursors to distress in many of the men they worked with. In line with the interpersonal theory of suicide (Van Orden et al., 2010), high prevalence of male suicide among regional communities may in part be explained by a heightened sense of thwarted belongingness flowing from physical isolation as well as misfitting with dominant ideals of masculinity (Oliffe et al., 2019a). Perceived burdensomeness was not strongly apparent in stakeholder accounts, possibly attributable to burdensomeness being an internalised and private construct. Data from *The Human Code Project* community survey will be able to verify rates of perceived burdensomeness in subsections of the community. Men high in burdensomeness may have been both less likely to be in contact with the stakeholders interviewed (e.g., healthcare workers), and less likely to disclose a sense of burdensomeness if they were. Further research with men in regional areas is required to examine the influence of burdensomeness on wellbeing in this population.

Rural and regional areas can present a narrowness of acceptable behaviours, particularly for young men, and provide few opportunities for those who move away from the traditional masculine norms (Coen et al., 2013; Creighton et al., 2017). Bolstering community connection by affording men increased diverse opportunities to expand their social connections may be a lynchpin for protective actions against male suicide behaviour in regional communities. The communal nature of regional areas can be capitalized, promoting formal and informal social networks and help giving, for example through peer support programs (Hirsch & Cukrowicz, 2014). Men’s Sheds^1^ were spoken of highly by stakeholders and offer a well-established government funded model of peer support shown to provide wellbeing benefits for men and increase sense of belonging (Waling & Fildes, 2017; Taylor et al., 2018). The success of the Men’s Shed model has been attributed to its fit within a masculine framework outside of typical healthcare contexts, where men are able to maintain independence through partaking in shared activities while creating emotional bonds and engaging in healthy social peer relationships. Men Shed’s however are normally targeted to, and engage older men. Initiatives aimed at enhancing belonging that are sensitized to men’s level of comfort in going beyond traditional masculine spaces are therefore needed for younger and middle-aged men. Structural, community based social initiatives may be more accessible and appealing to many men than individual approaches (e.g., individual therapy) as a means of coping with stress and increasing sense of community and belonging (Kutek et al., 2011).

Stakeholders reiterated the cultural importance of sporting clubs as a space where men gather and socialize, and as structures that are seen as key drivers of township cultures. Sport is an integral institution in the development and upholding of masculine ideals, through the valuing of athleticism, toughness and competitiveness (Burgess et al., 2003). Indeed, discussion of mental health from public sporting figures has been proposed to be potentially beneficial for young male help-seeking, by connecting vulnerability and help-seeking to aforementioned masculine ideals (Swann et al., 2018; Walton et al., 2019). In a more applied sense, sport settings provide men with the opportunity to socially connect in with community (Chamravi et al., 2020), and can be utilized as men-friendly community settings for health promotion messaging and mental health programs (Oliffe et al., 2019b). Programs of this nature that have been developed internationally (Dixon et al., 2019; Wilcock et al., 2021), and in Australia have aimed to promote early intervention and help-seeking (Vella et al., 2018; Vella et al., 2021), increase parental awareness of mental health (Hurley et al., 2020) and increase men’s self-efficacy, social connectedness, and leadership skills (Chamravi et al., 2020). The implementation of evidence-based programs like this into regional sporting organisations may be a key step forward to increasing belonging and connectedness for many men, and reducing stigma surrounding mental health help-seeking.

Many stakeholders spoke of the increased time stress for men working outside of the region with long commuting hours. While their workplaces may provide opportunities for social networking, having meaningful connections within their immediate community was seen as important. These men, likely within the age range of 30–60 years-old and often parents, represent a key gap in the provision of social groups for connection as they tend to fall outside of the target audience for sporting clubs aimed predominantly at younger men, and Men’s Sheds aimed at retirees. Understanding the needs of these men and providing opportunities for connection that are appealing and accessible within their time constraints is an important consideration. Additionally, equipping men with the social skills and confidence to seek out and maintain social connections with those they meet incidentally (i.e., through work or family commitments) may assist in overcoming barriers to prioritizing social connection for men who are time-poor. More work could be done to support and connect in specific groups of men, for example new fathers as they navigate changing roles, to ensure social connection is not lost to competing priorities. Importantly, communities should recognize that sporting clubs and other environments that embody traditional masculine qualities will not appeal to all demographics of men within a given region, and care should be taken to ensure choice in activities offered and provide safe spaces within communities for all men and boys. Consideration of the additional challenges faced by minority groups of men including LGBTQIA + men, culturally and linguistically diverse men, and Aboriginal and Torres Strait Islander boys and men is essential to delivering appropriate and identity affirming social structures for these groups. Further research is required to identify what these might look like in an inner regional community.

### Engaging With Men

As a way of overcoming the above concerns, stakeholders emphasized the need for a gender-sensitive approach to health promotion and behaviour change that recognizes the strengths and interests of individuals and works within a positive masculine framework to create reform. Authenticity in engagement with men and interventions coming from within the community were seen as key by stakeholders, who discussed the need to identify community role models who endorse positive masculine traits and who are respected, relatable and able to engage with the male population. Further, men sharing their lived experience of mental ill-health or stressful life events was seen as a powerful way to impart a message of courage, vulnerability and hope in the community. For example, prominent and respected sportsmen sharing their mental health stories can have a positive influence on men’s help seeking behaviours through repositioning speaking out about mental health as a social norm (Harding & Fox, 2015). Building capacity for men to be able to step into positive leadership roles within the community and providing spaces that support sharing lived experience may help to promote healthier traits in men and normalize help-seeking.

Stakeholders saw a need to tailor program service and delivery to be sensitized to men’s needs through the use of language and content. Health promotion messaging including labeling, advertising and content in community-based settings must be presented in ways that are accessible, easy to understand and sensitized to the specific needs of diverse audiences of men (Oliffe et al., 2019a). Within the content of programs, introducing mental health topics and language can help to reduce stigma by increasing men’s understanding and literacy. However, as mentioned of concern by stakeholders, the associated stigma means that many men do not respond to language promoting ‘mental health’ or associated terms. Working within their language framework and avoiding pathologizing terms may assist in attendance and engagement, for example the Veteran Transition Program uses familiar language such as ‘dropping your baggage’ and focusing on ‘release’ in counselling psychology programs with men (Cox et al., 2019). Further, care should be taken to avoid provocative language such as *toxic masculinity* that is likely to further disengage the population services are aiming to reach (VicHealth, 2020). Instead, stakeholders provided examples of positive framing which could be implemented, such as framing the vulnerability of help-seeking as courageous, although care should be taken to mitigate the risk of inadvertently reinforcing masculine norms through the assertion then that men who *don*’*t* seek help are outside of the norm and are displaying weakness.

Leaning on men’s sense of care for loved ones was viewed as a way to encourage caring for oneself. Elliott’s (2016) theory of *caring masculinities* talks to this point; elucidating the benefit of reframing traditional masculine values of protector and provider into prosocial care-oriented values that exist within relationships without male dominance. Upholding caring masculinities by contributing to child care work and work in the home can have a suite of benefits for men’s health including increased psychological wellbeing, longer life span, and improved intimate and nurturing relationships (Kimmel, 2010). The promotion of caring masculinities affords men with more flexibility in their masculine expression and may positively impact self-compassion and therefore comfort with help-seeking. In sum, health promotion efforts should seek to engage with men authentically, recognize the diverse influences of masculinities and provide opportunities for men in community to become positive role models and share their stories openly.

### Implications

The findings presented here uniquely describe the characteristics of an inner regional community which diverges from both rural and metropolitan spaces both in terms of culture, and in structures and services available. As migration out of major cities into surrounding regional and rural areas continues, an understanding of place-based conceptualisations of masculinities and mental health is required in order to ensure that these populations of men are supported. Notably, the findings around belonging offer unique insights into how these inner regional communities can constrain men’s social wellbeing, particularly in relation to the experiences of men who work away from their townships. With these additional challenges of geography and competing priorities in mind, the findings highlight the need to design community programs accordingly that understand and cater for these men’s needs. Importantly, this research demonstrates that consultation with community stakeholders is a vital step in understanding the needs of men in a given community, and the capacity of that community to meet those needs in order to facilitate better translation from research into practice. By specifically outlining ways in which services and programs within a regional community can work to better engage with and support men, we hope to provide insights that can lead to concrete and practical implementation strategies for effective community wellbeing and suicide prevention programs.

### Limitations and Future Directions

The present study has a number of limitations. First, the focus on stakeholder perspectives offers a third-party view into the challenges faced by local men and boys. While beneficial in its ability to draw from a diverse array of professional experiences and increase community engagement likely to enhance success of intervention implementation, future research into men’s direct experiences within the local community is required. Further, we recognize that a study of local masculinities enrolled majority female participants, and as above recommend that future research aims to unpack masculinity from local men’s personal perspectives. Female stakeholders’ insights however provide valuable contribution to the study as they live and work with boys and men in the community, and therefore impact and are impacted by men’s behaviours. Additionally, the specific concerns and needs of adolescent and younger men may not be well represented in the data due to the youngest participant being 33 years of age. We did not collect demographics other than age, gender identity and professional sector, limiting our ability to characterise and describe the sample. While interview schedules focused on questions related to professional opinions and experiences working with boys and men in the Macedon Ranges, it is likely that participant responses were influenced by their own personal preconceptions of and experiences with masculinity. Participants’ (particularly male participants’) own embodiment of masculinities are reflected throughout the findings, as they often switched between providing first person and third person narratives to explain their perspectives. There were varying levels of experience with or knowledge of masculinity and it’s impacts on mental health dependent on the participant’s sector and role. Some participants expressed that they had previously not put much thought into masculinity or ‘what it means to be a man’, while others actively worked to separate themselves from what they saw as dominant and harmful norms. The influence of participants personal ideas of masculinity should be taken into consideration when interpreting the findings. Stakeholders in this study were recruited through contacts of the Project Working Group. As such, the current sample may not be representative of stakeholder and service provider views more widely in the region. Having said that, participants worked across community sectors and were in contact with a diverse range of local men and boys in different facets. Given the relatively close proximity of the Macedon Ranges to the large urban center of Melbourne, results from the present study many not reflect views of stakeholders from more remote rural settings. Further, research should also seek to ensure the voices of rural and regional stakeholders from multicultural backgrounds are represented regarding to risk and protective factors for men’s suicide.

## Conclusion

The findings of this study describe stakeholder perspectives of the challenges for wellbeing faced by men in a regional community, and opportunities for intervention. Garnering stakeholder insights allowed for a diverse range of professional experiences within the research to inform better design, implementation and retention of future community-driven initiatives to support men and boys. Results indicated that community-based suicide prevention strategies must focus on increasing men’s sense of belonging within their communities by expanding the range of opportunities offered for healthy social interaction and also should promote a masculinity that allows for improved social connection and help-seeking for mental health problems when needed. Understanding the specific needs of men in an inner regional community that differs culturally and geographically from both urban and rural environments is a vital step towards reducing suicide and other harm-behaviours in men.

## Data Availability

The datasets presented in this article are not readily available because of ethical restrictions. Requests to access the datasets should be directed to Simon Rice, simon.rice@orygen.org.au.

## Footnotes

1 Men’s Sheds are community-based nonprofit organisations accessible to all men within a community with the primary purpose of providing a safe and friendly environment for men to work on meaningful projects in the company of other men (see https://mensshed.org/what-is-a-mens-shed/). Aimed at advancing the health and well-being of their male members, there are over 1,000 Men’s Sheds in Australia and increasing numbers worldwide.
